# Arthroscopic surgery for the treatment of a geriatric bipartite talus: A case report

**DOI:** 10.1097/MD.0000000000047195

**Published:** 2026-01-09

**Authors:** Xianmei Xiong, Meng Chen, Guojie Lin, Hao Peng, Qinmeng Yang, Changqing Pan, Weijian Chen, Zhong Yang, Xiaoyong Fu

**Affiliations:** a Department of Foot and Ankle Surgery, Guangzhou Orthopedic Hospital, Guangzhou, Guangdong, China; b Department of Medical Education, Guangzhou Orthopedic Hospital, Guangzhou, Guangdong, China.

**Keywords:** ankle pain, arthroscopic surgery, bipartite talus, geriatric patient, rare case

## Abstract

**Rationale:**

Bipartite talus is a rare anatomical variation characterized by the division of the talus into 2 distinct fragments by a cartilaginous or fibrocartilaginous interface. Clinically, bipartite talus primarily manifests as posterior ankle pain accompanied by joint stiffness and start-up pain, necessitating differentiation from the posterior talus triangle. Currently, reported cases of bipartite talus are scarce, particularly among elderly patients, and treatment protocols remain scarcely reported.

**Patient concerns:**

A 60-year-old female patient presented with persistent pain and limited mobility in the posterior aspect of her left ankle over the past 2 years, with symptoms significantly worsening 1 year prior. She presented no history of trauma or infection. Physical examination revealed mild posterior swelling, tenderness in the posterolateral region, and pain on initiation of movement. There was mild limitation of plantar flexion in the left ankle joint. Imaging examinations including x-ray, computed tomography and magnetic resonance imaging demonstrated a fairly large bone fragment.

**Diagnoses:**

Bipartite talus (left side).

**Interventions:**

Perform arthroscopic surgery on the patient.

**Outcomes:**

The patient’s pain and joint mobility limitations showed significant improvement.

**Lessons:**

Arthroscopic technology can be used as an effective means of treating traumatic bipartite talus with minimal trauma and rapid recovery. However, its long-term efficacy needs to be verified by a larger sample size and longer follow-up.

## 1. Introduction

Talus bisection is a rare anatomical variation characterized by the division of the talus into 2 distinct fragments by a cartilaginous or fibrocartilaginous interface, as opposed to the Os trigonum. A triquetral process is a benign posterosuperior process,^[1]^ but a bipartite talus involves a split plane anterior to the posterior process, resulting in separate talar bodies and a posterior process fragment, often with damage to the subtalar joint^.^ First clinically reported in 1975,^[2]^ <20 cases have been described,^[3]^ mainly occurring in adolescents and young adults, presenting with chronic posterior foot pain, restricted subtalar motion, or neuropathy.

The etiology of the bipartite talus remains controversial. The current mainstream embryological theory is that there is a talus ossification center at the seventh month of pregnancy, but if a secondary ossification center occurs when the bone is immature, it will cause the talus to be divided.^[3,4]^ It is worth noting that the bipartite talus may also be related to the internal torsion of the tibia and the hypothetical stress caused by manual therapy in infancy, which indicates that biomechanics contributes to the separation of fracture fragments.^[5]^ There are diagnostic challenges in differentiating a bipartite talus from post-traumatic pseudarthrosis or an atypical Os trigonum, and advanced imaging computed tomography (CT)/magnetic resonance imaging (MRI) and histological analysis are required to confirm joint involvement and rule out degenerative changes. Treatment options range from conservative management to fragment removal, fixation or arthrodesis, with surgical management dependent on the size of the fracture fragment and joint degeneration. Recent reports have highlighted new presentations such as tibial nerve entrapment within the tarsal canal, expanding the clinical spectrum. These 2 case reports highlight the diagnostic complexity and treatment nuances of talar division, emphasizing their awareness of avoiding misdiagnosis and optimizing outcomes.^[6,7]^

Given that previous case studies have mainly been conducted on adolescents and young adults, we report a case of arthroscopic surgical removal of bone fragments in an elderly bipartite talus, which may provide some reference for the treatment of elderly bipartite talus.

## 2. Case report

### 2.1. General information

The patient is a 60-year-old woman. Although the patient is relatively elderly, she only began to experience pain in her left ankle joint 2 years ago, along with limited mobility. The pain worsened significantly 1 year ago, and the pain is mainly located at the back of the ankle joint (not the subtalar joint). The patient reports no history of trauma or infection. After >3 months of conservative treatment, including wearing walking boots, taking non-steroidal anti-inflammatory drugs, and physiotherapy, the symptoms improved occasionally but were not completely resolved.

### 2.2. Physical examination

No obvious abnormalities were found during the general physical examination. The left ankle joint was not obviously deformed, with slight swelling at the posterior side, tenderness at the posterolateral side, and triggering pain. There was mild limitation of plantar flexion of the left ankle joint, and the mobility of the talocrural joint was normal. There were no abnormalities in the sensory or vascular examinations. The American Orthopaedic Foot and Ankle Society score was 54 points.

### 2.3. Imaging examination

**Figure 1.**
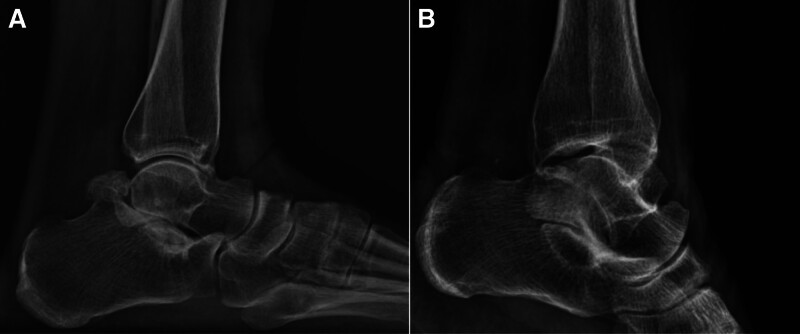
(A) Preoperative lateral radiograph showing a bipartite talus. (B) Postoperative lateral radiograph showing the bipartite talus removed.

**Figure 2.**
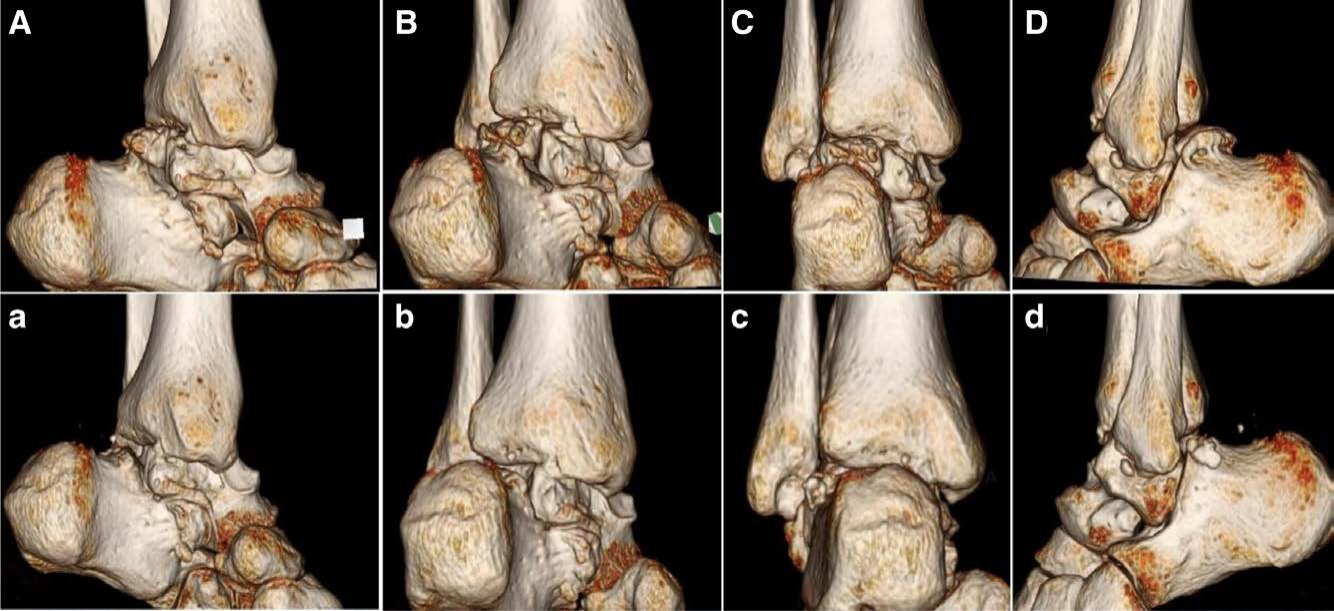
(A–D) Preoperative CT 3D scan images clearly show the bipartite talus and its size. (a–d) Postoperative CT 3D scan images show that the bipartite talus has been basically removed. CT = computed tomography.

**Figure 3.**
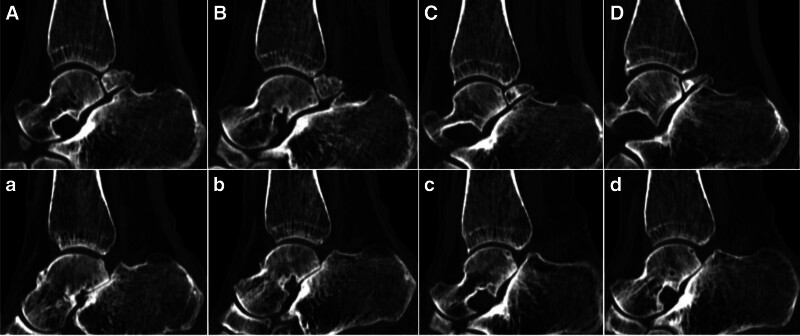
(A–D) Preoperative sagittal CT scan radiograph showing a bipartite talus with concomitant degenerative changes of the subtalar joint. (a–d) Preoperative sagittal CT scan radiograph showing the basic removal of the bipartite talus. CT = computed tomography.

**Figure 4.**
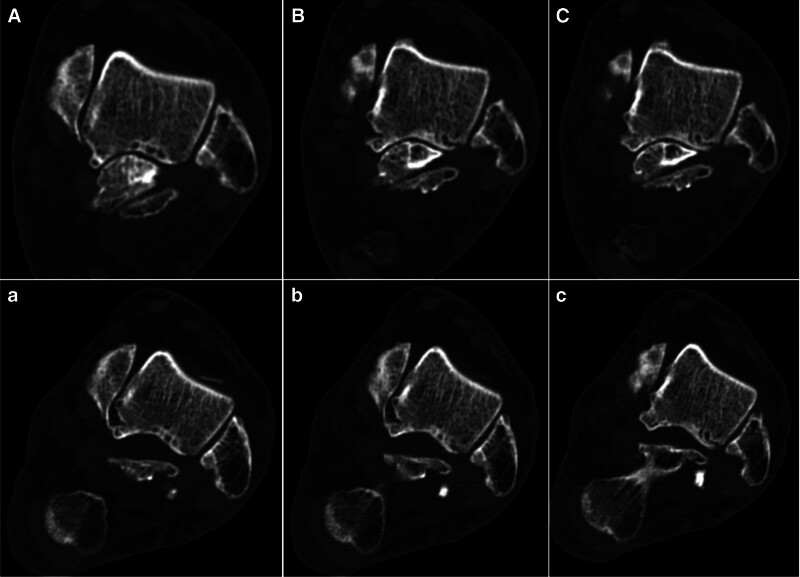
(A–C) Preoperative axial CT scan images show that the diaphyseal radius accounts for about 1/4. (a–c) Postoperative axial CT scan images show that the bipartite talus has been basically resected. CT = computed tomography.

**Figure 5.**
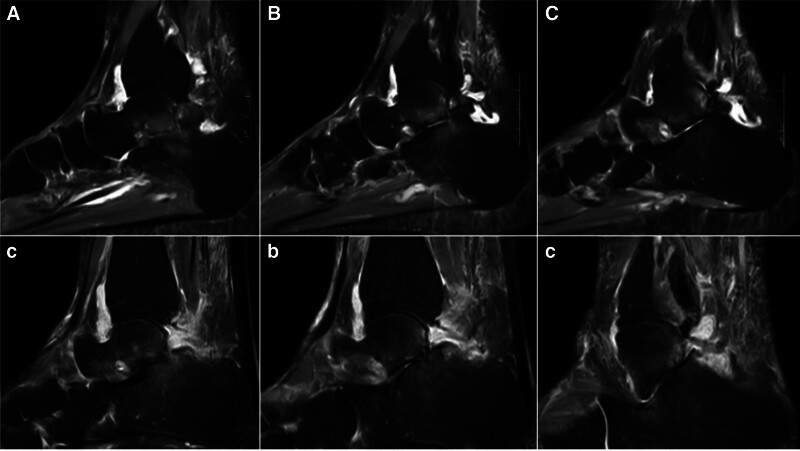
(A–C) Preoperative sagittal MRI scan images show relatively severe bone marrow edema signals in the anterior ankle joint and posterior talus, and relatively mild bone marrow edema signals in the subtalar joint. (a–c) Postoperative sagittal MRI scan images shows that the bipartite talus has been almost completely resected. CT = computed tomography, MRI = magnetic resonance imaging.

A preoperative weight-bearing lateral radiograph (Fig. 1) shows a fairly large bone fragment behind the talus. The preoperative CT scan images (Figs. 2–4) show that the posterior bone fragment accounts for about a quarter of the talar fossa. The preoperative MRI images (Fig. 5) show that relatively severe bone marrow edema signals in the anterior ankle joint and posterior talus, and relatively mild bone marrow edema signals in the subtalar joint.

### 2.4. Surgical intervention

**Figure 6.**
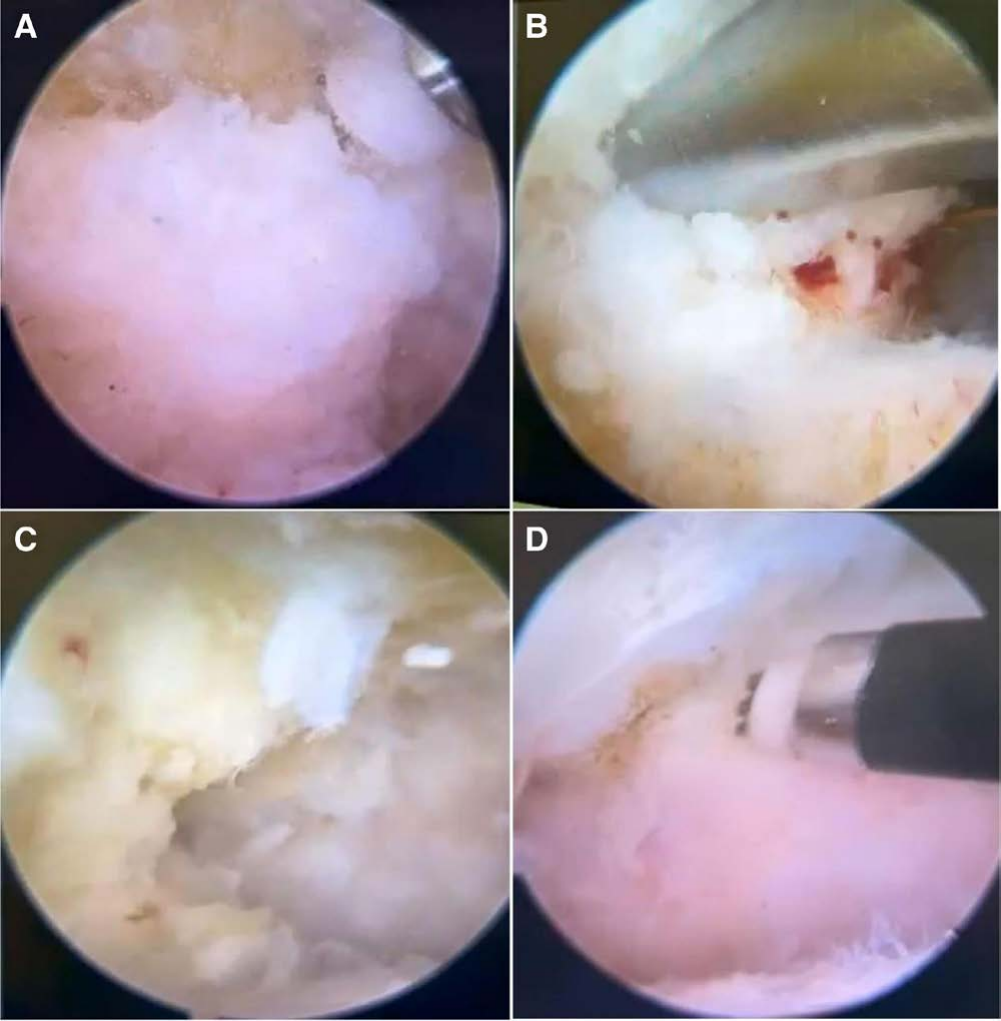
Arthroscopic view. Figure (A) shows the location and size of the bipartite talus; (B) shows the bone fragment being clamped; (C) shows partial resection of the bone fragment; (D) shows complete resection of the bone fragment.

The lateral and medial Achilles tendon approaches were used,^[8]^ with the plane located 1 cm above the tip of the outer malleolus. After the arthroscopic lens was inserted, a comprehensive exploration of the joint was performed to explore the posterior anatomical structures. A rasp was used to remove the posterior bony fragment (Fig. 6), and a plane knife was used to remove scar tissue and hyperplastic ligament scar tissue. Intraoperative x-rays were taken to assess the excision, and the incision was then rinsed and closed layer by layer.

### 2.5. Postoperative treatment

ankle dorsiflexion and plantar flexion can be started on the third day after the operation, and weight-bearing walking is prohibited within 2 weeks after the operation. After 2 weeks after the operation, gradually start weight-bearing exercises with the assistance of an inflatable boot. Remove the inflatable boot from the sixth week after the operation, and at the same time inform the patient that they must not participate in sports for 2 months. Follow-up visits are scheduled for 3 months, 6 months, and 1 year after the operation.

## 3. Results

Postoperative x-rays, CT and MRI (Figs. 1–5) and intraoperative arthroscopic images (Fig. 6) showed that the bone fragments had been basically removed. The patient did not experience any complications such as infection or nerve damage after the operation. At the 3-month follow-up, the patient’s pain symptoms had improved significantly, and the limitation of movement had improved significantly. Six months after the operation, the patient reported no recurrence of symptoms and an American Orthopaedic Foot and Ankle Society score of 85 points. At the 1-year postoperative follow-up, the patient reported only mild discomfort after prolonged walking, and no obvious signs of subtalar joint instability were observed.

## 4. Discussion

In clinical diagnosis, bipartite talus is most easily confused with Os trigonum syndrome. First, in terms of clinical symptoms, bipartite talus is mainly manifested as pain in the posterior ankle joint with joint stiffness, accompanied by triggering pain, while Os trigonum syndrome is mainly manifested as posterior ankle impingement syndrome, with obvious pain during plantar flexion (such as the “ballet toe action”). Second, both generally have no history of trauma, However, some patients with bipartite talus have a history of repeated ankle sprains, but these may not be accompanied by high-energy trauma. Symptoms of Os trigonum syndrome tend to worsen due to acute injury. Bipartite talus is often accompanied by degenerative joint changes, mainly seen in the pseudarthrosis area and adjacent joints (such as damage to the cartilage of the inferior talus joint). Os trigonum syndrome is mostly not associated with degenerative joint changes.^[9]^ On imaging, on the first x-ray (lateral view), the bipartite talus generally shows a large (>1 cm) bone fragment at the posterior ankle, which may be accompanied by joint surface degeneration, while the posterior Os trigonum generally presents as a small triangular bone fragment at the posterior ankle with clear boundaries. On CT and MRI, the bipartite talus bone fragment is generally connected to the talus body by a pseudarthrosis-like connection, accompanied by bone marrow edema and articular cartilage degeneration. It often involves the posterior joint surface of the talonavicular joint on CT/MRI. while the bone fragment in the s trigonum syndrome is generally connected to the talus by fibrous cartilage without bony fusion, with clear boundaries, and is located only at the back of the ankle, without contacting the main joint surface^.[10]^

Etiologic theories of bipartite talus remain controversial. The current mainstream theory for the cause of the bipartite talus is the abnormal embryonic development hypothesis, which states that the fusion of the ossification center failed.^[3]^ They believe that it was formed by the fusion of the “tibiale” and “intermedium” (Čihak embryological research), and the bipartite talus is described as a developmental abnormality formed by aseptic separation before fusion. It is mainly located at the junction of the posterior process of the talus and the body of the talus. Another theory is the currently controversial trauma-related hypothesis,^[5]^ which suggests that the bipartite talus is caused by an injury with axial compound before puberty or a fracture with shear of the posterior talar articular surface in a violent position of hyperflexion. A third theory is the pathological pseudarthrosis theory,^[2]^ in which the bipartite talus is formed due to abnormal biomechanical loading on the posterior talus in childhood, most commonly seen with repeated posterior ankle impingement (such as ballet ankle plantar flexion overload) or tibial torsion deformity. In addition, there are theories that bipartite talus is related to endocrine effects,^[11]^ and some scholars believe that it is an atavistic phenomenon.^[12]^

The treatment of bipartite talus is still controversial at present, but most of the cases described in previous studies did not show significant improvement after more than 6 months of conservative treatment, and surgical treatment was chosen. At present, the main surgical treatment options for bipartite talus are excision, fixation and excision with arthrodesis. In the first case of bipartite talus reported in the literature,^[2]^ they resected the bipartite talus. Subsequently, Matthew et al^[5]^ reported 2 cases of patients aged 18 and 16, who both underwent surgical removal of bone fragments, but he decided whether to perform arthrodesis surgery based on the stability of the subtalar joint after surgery. In the end, the symptoms of both cases improved significantly. Mann and Myerson et al^[11]^ performed surgical removal of bone fragments in all 5 cases aged 13 to 16 years studied. At follow-up, complete recovery of symptoms was found in 3 cases, while 2 cases still had symptoms after exercise. Thiel et al^[13]^ reported a case of a patient with a bipartite talus who had received a single screw for fixation. After surgical treatment, the patient had no pain and was not limited in activity. However, imaging showed that the fixed bone fragment had not healed 9 months after surgery. Rose et al^[14]^ studied 5 cases aged 11 to 55 years. He implemented different surgical plans for patients based on the size of the bone fragment. For bone fragments smaller than 20% of the surface area of the talar navicular joint, surgical excision was performed. For bone fragments larger than 20% of the subtalar joint surface, options include fixation of the fragment or excision with arthrodesis. Among these, 1 case of fragment fixation presented with nonunion, but the results showed that the symptoms had improved and subsided. Zwiers et al^[3]^ first reported 2 cases that received arthroscopic surgery, in which they performed osteotomy under arthroscopy and fixed the osteotomy with screws on the body of the talus. Both the short-term and long-term follow-ups showed good results. In addition, 2 case report in the literature reported 2 cases of bipartite talus with tarsal tunnel syndrome. After the bone fragments were removed, the symptoms of nerve compression improved significantly.^[6,7]^

In summary, among the 17 previously reported surgical cases for bipartite talus, the vast majority (11 cases) received excision surgery alone, a minority (4 cases) received fixation surgery, and a very small number (2 cases) received excision with arthrodesis. Postoperative symptoms improved significantly in most excision cases, while nonunion occurred in half (2 cases) of the fixation surgeries. Arthrodesis is generally a relatively invasive procedure, carrying risks of wound complications and nonunion. It also results in substantial loss of joint mobility and may accelerate degeneration in adjacent joints.^[15,16]^

In our case, the patient was a 60-year-old woman whose symptoms developed over a 2-year period. After more than 6 months of conservative treatment with no significant improvement, she ultimately elected to receive surgical treatment. Regarding the choice of surgical protocol, we considered that although the patient had a certain degree of osteoarthritis, the joint degeneration was not severe, the subtalar joint involvement was not extensive, and the patient did not experience significant pain in this joint. Most importantly, the patient had specific requirements for joint mobility and expressed reluctance toward major open surgery (which would make arthrodesis relatively difficult to perform minimally invasively). Therefore, we ultimately opted for a solution involving only the excision of the bone fragment, and we performed the procedure using arthroscopic minimally invasive surgery.

To the best of our knowledge, this is the first reported case of arthroscopic surgery for bilateral talus in an elderly patient. Extensive literature indicates that arthroscopic treatment offers advantages over open surgery in numerous aspects, as it results in less soft tissue damage, minimal trauma, faster recovery, shorter hospital stays, and smaller scars.^[17]^ This makes it particularly suitable for elderly patients. The posterior ankle approach (medial and lateral to the Achilles tendon) has been demonstrated to be a safe and effective treatment option for posterior ankle pathologies.^[18]^

With the increasing number of reported cases of bipartite talus, our understanding of this condition has deepened. Combined with our current clinical case, we suggest that treatment for bipartite talus should be individualized based on the patient’s symptoms, the location and size of the fragment, the extent of joint involvement, the degree of osteoarthritis, and the patient’s specific requirements for joint mobility and surgical trauma. This approach ensures that surgical goals align with the patient’s unique circumstances and overall health status.

There are some limitations to our study. First, as we only had 1 case of arthroscopic surgery to remove bone fragments, we cannot draw universal conclusions. Second, we did not have any cases of open surgery, so we could not evaluate its advantages and disadvantages compared to traditional open surgery. In addition, the follow-up period was relatively short (only 1 year), so we could not evaluate the long-term efficacy. Therefore, it is recommended that future multicenter studies or case series (if more such cases are encountered) be conducted, enabling comparative research between minimally invasive and open surgical approaches, while also securing longer-term follow-up to confirm the generalizability of the findings.

## 5. Conclusion

We report for the first time the case of a patient with an elderly bipartite talus who was treated with arthroscopic surgery. Short-term follow-up showed that the patient’s symptoms improved significantly and his function recovered well. Cases of bipartite talus are relatively rare, and in clinical practice, it is necessary to differentiate them from tripartite talus and post-traumatic pseudarthrosis through CT/MRI. Arthroscopic surgery is a good alternative.

## Acknowledgments

We thank the patient and her family members for their contributions to this study.

## Author contributions

**Data curation:** Guojie Lin, Weijian Chen.

**Investigation:** Qinmeng Yang.

**Resources:** Changqing Pan.

**Supervision:** Xiaoyong Fu.

**Validation:** Hao Peng.

**Visualization:** Meng Chen.

**Writing – original draft:** Xianmei Xiong.

**Writing – review & editing:** Zhong Yang, Xiaoyong Fu.
